# 5-LO-derived LTB4 plays a key role in MCP-1 expression in HMGB1-exposed VSMCs via a BLTR1 signaling axis

**DOI:** 10.1038/s41598-021-90636-2

**Published:** 2021-05-27

**Authors:** Jong Min Choi, Seung Eun Baek, Ji On Kim, Eun Yeong Jeon, Eun Jeong Jang, Chi Dae Kim

**Affiliations:** 1 Department of Pharmacology, School of Medicine, Pusan National University, Yangsan, Gyeongnam 50612 Republic of Korea; 2 Gene and Cell Therapy Research Center for Vessel-Associated Diseases, Pusan National University, Yangsan, Gyeongnam 50612 Republic of Korea; 3 Research Institute for Convergence of Biomedical Science and Technology, Pusan National University Yangsan Hospital, Yangsan, Gyeongnam 50612 Republic of Korea

**Keywords:** Physiology, Cardiology, Medical research

## Abstract

Monocyte chemoattractant protein-1 (MCP-1) plays an important role in initiating vascular inflammation; however, its cellular source in the injured vasculatures is unclear. Given the importance of high mobility group box 1 (HMGB1) in tissue injury, we investigated the role of vascular smooth muscle cells (VSMCs) in MCP-1 production in response to HMGB1. In primary cultured rat aortic VSMCs stimulated with HMGB1, the expression of MCP-1 and 5-lipoxygenase (LO) was increased. The increased MCP-1 expression in HMGB1 (30 ng/ml)-stimulated cells was significantly attenuated in 5-LO-deficient cells as well as in cells treated with zileuton, a 5-LO inhibitor. Likewise, MCP-1 expression and production were also increased in cells stimulated with exogenous leukotriene B4 (LTB4), but not exogenous LTC4. LTB4-induced MCP-1 expression was attenuated in cells treated with U75302, a LTB4 receptor 1 (BLTR1) inhibitor as well as in BLTR1-deficient cells, but not in 5-LO-deficient cells. Moreover, HMGB1-induced MCP-1 expression was attenuated in BLTR1-deficient cells or by treatment with a BLTR1 inhibitor, but not other leukotriene receptor inhibitors. In contrast to MCP-1 expression in response to LTB4, the increased MCP-1 production in HMGB1-stimulated VSMC was markedly attenuated in 5-LO-deficient cells, indicating a pivotal role of LTB4-BLTR1 signaling in MCP-1 expression in VSMCs. Taken together, 5-LO-derived LTB4 plays a key role in MCP-1 expression in HMGB1-exposed VSMCs via BLTR1 signaling, suggesting the LTB4-BLTR1 signaling axis as a potential therapeutic target for vascular inflammation in the injured vasculatures.

## Introduction

Vascular smooth muscle cells (VSMCs) are known to play important roles in the progression of various vascular diseases, including vascular remodeling. VSMCs exhibits distinct proliferative and migratory abilities and produces proinflammatory cytokines by changing their phenotypes from contractile to synthetic one^1,2^. In our previous study, VSMCs produced IL-1β in response to high mobility group box 1 protein (HMGB1), a prototypical damage-associated molecular pattern (DAMP)^3^. Thus, VSMCs have been implicated as an active player in the development of proliferative vascular diseases associated with vascular injury^4,5^.

In vascular inflammation such as atherosclerosis, leukotriene B4 (LTB4), a product of 5-lipoxygenase (5-LO) in the arachidonic acid metabolism, has been implicated as a pivotal proinflammatory mediator^6^. In the injured vasculatures of mice deficient of 5-LO activating protein (FLAP), neointima formation was markedly attenuated by reduction of inflammatory cytokines release from FLAP-deficient macrophages^7^. In addition, a pivotal role for 5-LO in the pathogenesis of vascular remodeling diseases has been demonstrated in our previous studies^8–10^. Although recent studies have suggested strong links between 5-LO-derived metabolites and vascular inflammation^11–13^, the precise molecular mechanisms are unclear.

The accelerated recruitment of circulating monocytes into the injured vasculatures is an early event in vascular inflammatory responses^14^. The transmigration and infiltration of monocytes into the injured vessel wall are mediated by chemokines such as monocyte chemoattractant protein-1 (MCP-1), one of the most potent chemoattractant cytokines for monocytes^15,16^. In addition to monocytes, MCP-1 plays a crucial role in the recruitment of memory T cells and dendritic cells to the sites of tissue injury, indicating its importance in tissue inflammation^17,18^. The importance of MCP-1 signaling pathway has also been reported in neointimal formation and vascular inflammation after perivascular injury^19^.

Although MCP-1 is a key player in the initiation and progression of vascular inflammation^20,21^, the cellular source of MCP-1 in the injured vasculatures is poorly understood. Given the importance of HMGB1 in vascular inflammation in the injured vasculatures, we investigated MCP-1 production and related molecular mechanisms in HMGB1-stimulated VSMCs to determine the active participation of VSMCs in vascular inflammation in the injured vasculatures.

## Materials and methods

### Ethics statements and animals

All experimental procedures were performed in accordance with the Guide for the Care and Use of Laboratory Animals published by the US National Institute of Health (NIH Publication No.85-23, 2011 revision), and all experimental protocols were approved by the Pusan National University Institutional Animal Care and Use Committee. As reported previously^10^, the genotyping of 5-LO KO and BLTR1 KO mice was performed by polymerase chain reaction (PCR) using a provided protocol by Jackson Laboratories (Harlan Nossan, Italy). Sprague-Dawley rats and wild-type (WT) control mice (C57BL/6J) were purchased from Charles River Breeding Laboratories (Kingston, NY, USA) and Jackson Laboratories, respectively. The study was carried out in compliance with the ARRIVE guidelines.

### Chemicals and antibodies

Recombinant human HMGB1 antibody was purchased from R&D System Inc. (Minneapolis, MN, USA). Zileuton (( ±)-*N*-hydroxy-*N*-(1-benzo[b]thien-2-ylethyl)urea) was purchased from Sigma-Aldrich Inc. (St. Louis, MO, USA). The MCP-1 antibody was purchased from Invitrogen (Carlsbad, CA, USA) and BLTR1 antibody was purchased from Biorbyt (Cambridge, UK). 5-LO and β-actin antibodies were purchased from Santa Cruz Biotechnology Inc. (Beverly, MA, USA). LTB4, LTC4, BLTR1 inhibitor (U75302), BLTR2 inhibitor (LY255283), LTC4 receptor (CysLTR) 1 inhibitor (Montelukast) and CysLTR2 inhibitor (HAMI3379) were purchased from Cayman Chemical Inc. (Ann Arbor, MI, USA). Horseradish peroxidase (HRP)-conjugated IgG antibody (Santa Cruz Biotechnology Inc.) was used as secondary antibody.

### Cell culture

Sprague-Dawley rats (7 weeks old, male) and mice (7 weeks old, male) were euthanized by CO_2_ inhalation, and then dissected to separate the thoracic aortas. The excised aortas were explanted in a cell culture dish as previously described^22^. Primary VSMCs were cultured in a cell culture dish containing Dulbecco’s modified Eagle’s medium (DMEM) (Gibco BRL, Grand Island, NY, USA) with 10% fetal bovine serum (FBS) (Gibco BRL). Cells were maintained in DMEM with 10% FBS and antibiotic–antimycotic solution (Gibco BRL) at 37 ℃.

### Western blot analysis

VSMCs lysates extracted were prepared in ice lysis buffer (Thermo Fisher Scientific, Rockford, IL, USA), and Western blot analysis were performed by the method previously described^22^. Equal amounts of the extracted protein were separated on 10–12% sodium dodecyl sulphate (SDS)-polyacrylamide gels under reducing conditions, and then transferred onto nitrocellulose membranes (Amersham-Pharmacia Biotech, Piscataway, NJ, USA). The membranes were blocked with 5% skim milk in Tris-buffered saline with Tween-20 (TBST) for 2 h and then incubated overnight with anti-MCP-1 (1:1000), 5-LO (1:1000) and BLTR1 (1:1000) in 5% skim milk. Incubated membranes were then washed with TBST, and incubated with HRP-conjugated secondary antibody for 2 h. Blots were developed using the Enhanced Chemiluminescence (ECL) Western blotting detection reagents (Amersham-Pharmacia Biotech) and utilizing image capturing software (Amersham-Imager 680, version. 2.0.). Membranes were re-blotted using anti-β-actin (1:1000) as an internal control.

### Small Interfering RNA (siRNA) preparation and transfection

As described in our previous report^3,8^, the siRNAs for 5-LO and BLTR1, and scrambled siRNA for the negative control (Cat no. SN-1003) were designed and synthesized using a AccuTarget siRNA construction kit purchased from Bioneer (Daejoen, ROK). The 5-LO siRNA-targeting sequence was 5′-CUG UUC AUC AAU CGC UUC A-3′ (forward) and 5′-UGA AGC GAU UGA UGA ACA G-3′ (reverse); for BLTR1 siRNA-targeting, 5′-GUC ACU AUG UCU GUG GAG U-3′ (forward) and 5′-ACU CCA CAG ACA UAG UGA C-3′ (reverse). All siRNA molecules were transfected through holes in the cell membrane using Lipofectamine 2000 (Invitrogen) according to the manufacturer’s protocol.

### Enzyme-linked immunosorbent assay (ELISA)

The cultured rat VSMCs stimulated with HMGB1, and then LTB4 production in the culture media was measured using a rat ELISA kit (Enzo life science, Farmingdale, NY, USA). The plate was read at 405 nm. In this study, MCP-1 production was determined in the culture media of rat VSMC and VSMCs from WT, 5-LO- and BLTR1-deficient mice. MCP-1 production in HMGB1- or LTB4-stimulated VSMCs was measured using rat MCP-1 ELISA kit (Abcam, Cambridge, MA, USA) and mouse MCP-1 ELISA kit (R&D Systems) according to manufacturer’s protocols. The plate was read at 450 nm.

### Statistical analysis

Results were expressed as means ± SEM. Student’s *t*-test or one-way analysis of variance (ANOVA) followed by Dunnett’s multiple comparison test was used to determine the significances of experimental results. Analysis of data was quantified using GraphPad Prism 5, version. 5.01. software (GraphPad Software, USA). Statistical significance was accepted for *p* values < 0.05.

## Results

### HMGB1 increases MCP-1 expression in VSMCs

**Figure 1:**
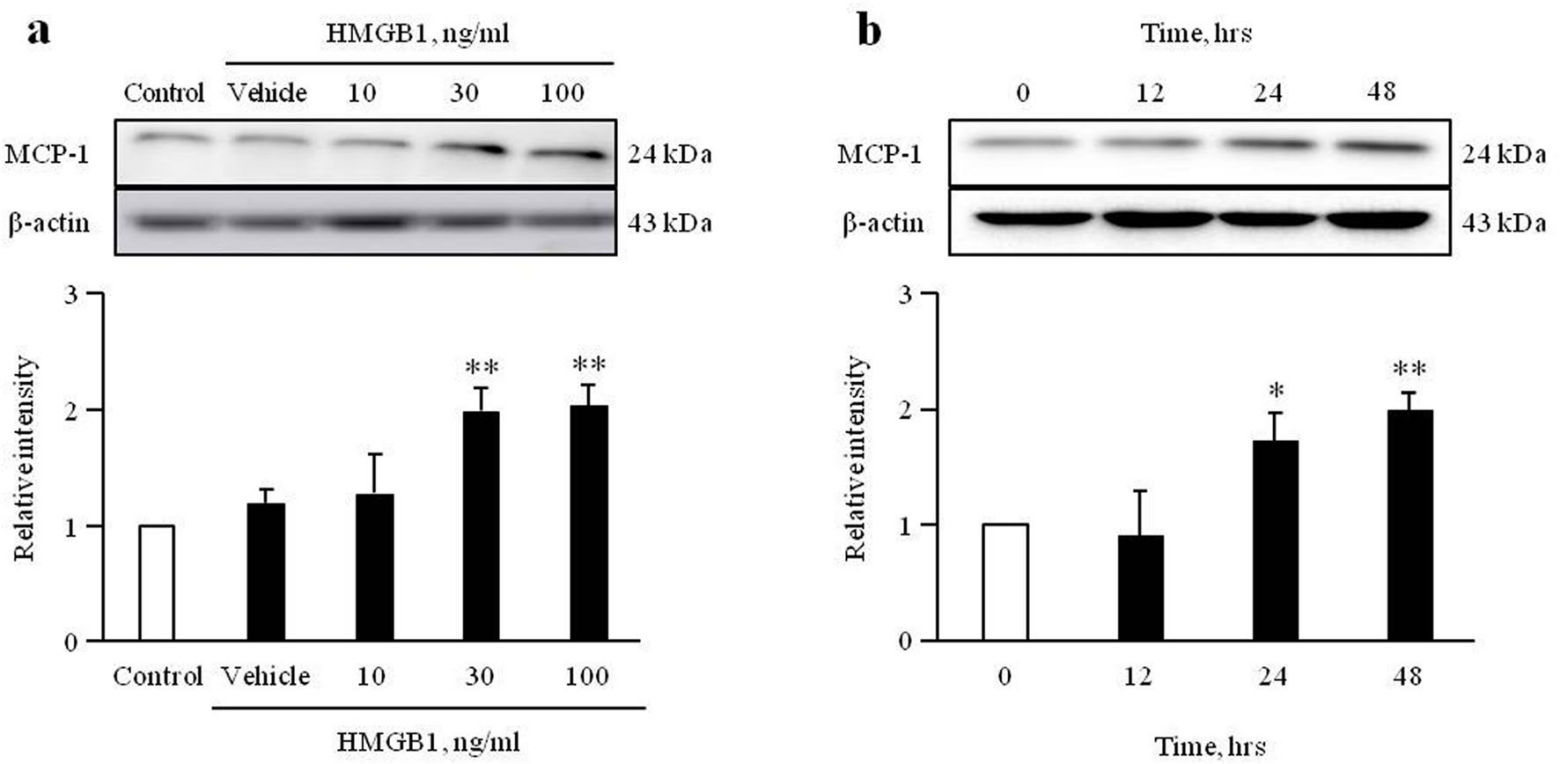
Role of HMGB1 on MCP-1 expression in VSMCs. Rat aortic VSMCs were treated with HMGB1 (0 to 100 ng/ml) for 48 h (**a**) or HMGB1 (30 ng/ml) for 0 to 48 h (**b**). The MCP-1 expression levels were determined by Western blotting. β-actin was used as an internal control. Relative intensities were expressed as the mean ± SEM of 3–4 independent experiments. *p < 0.05 and **p < 0.01 vs. corresponding value in control or 0 h. Image analysis was performed using the Amersham-Imager 680, ver. 2.0. software (https://www.cytivalifesciences.com/). Signal was quantified with GraphPad Prism 5, ver. 5.01. software (https://www.graphpad.com/). Cropped gels were displayed and full-length blots/gels are presented in supplementary Fig. S1.

To investigate the effect of HMGB1 on MCP-1 expression in VSMCs, cells cultured from rat thoracic aorta were serum starved for 24 h, and then stimulated with HMGB1. As shown in Fig. 1 and Supplementary Fig. S1, MCP-1 expression in HMGB1 (30 ng/ml)-treated cells increased at 24 ~ 48 h. At this time of HMGB1 stimulation, MCP-1 expression in VSMCs was increased in a dose-dependent manner. In this experiment, dose-dependency was observed up to HMGB1 concentration of 30 ng/ml, and hence, this concentration was selected for subsequent experiments.

### HMGB1 increases 5-LO activity and expression in VSMCs

**Figure 2:**
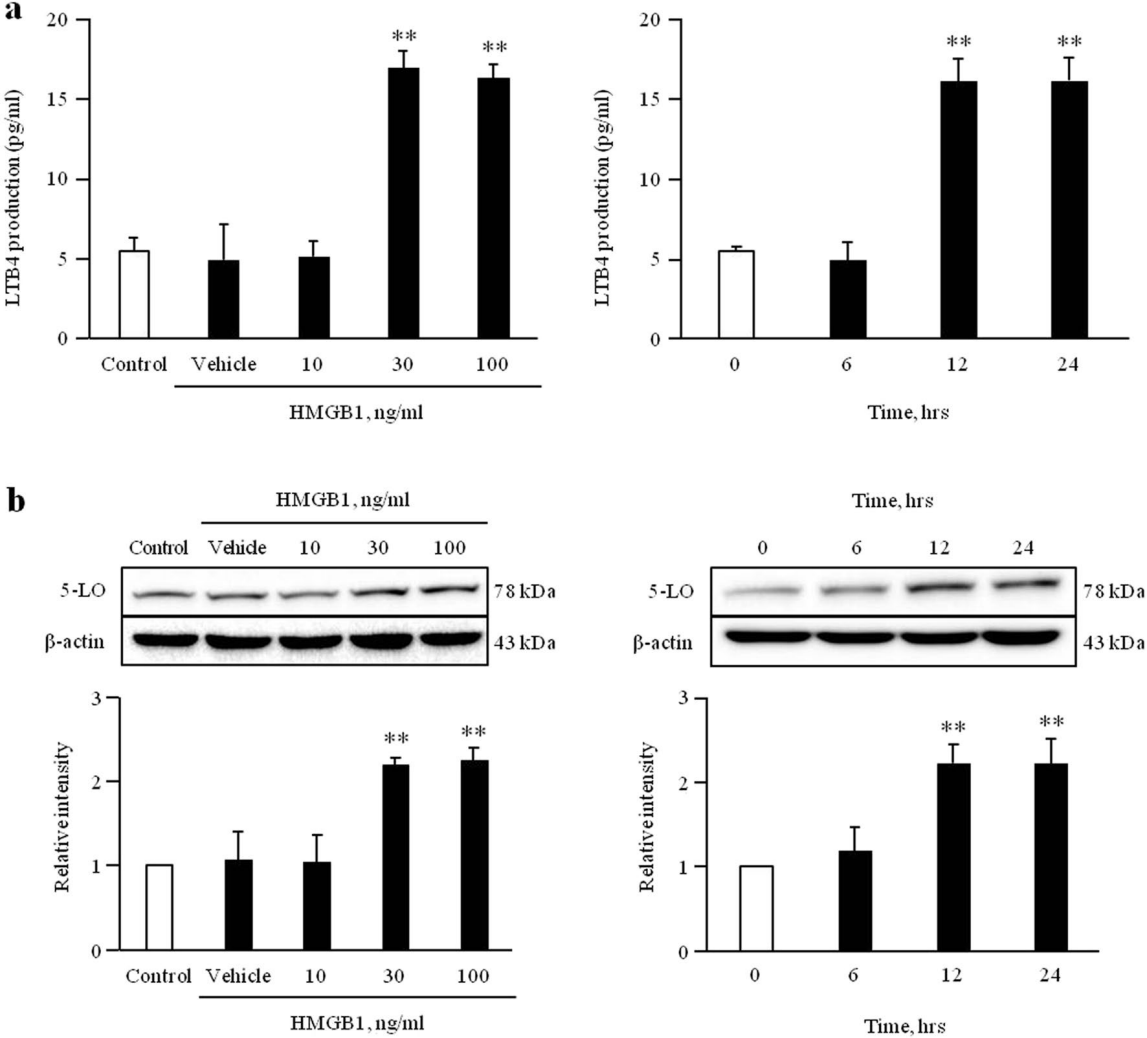
Role of HMGB1 on 5-LO activity and expression in VSMCs. VSMCs were treated with HMGB1 (0 to 100 ng/ml) for 12 h or HMGB1 (30 ng/ml) for 0 to 24 h. (**a**) LTB4 production was measured in the cell culture media, and results were expressed as the mean ± SEM of 3 independent experiments. **p < 0.01 vs. corresponding value in control or 0 h. (**b**) The 5-LO expression levels were determined by Western blotting. β-actin was used as an internal control. Relative intensities were expressed as the means ± SEMs of 4–5 independent experiments. **p < 0.01 vs. corresponding value in control or 0 h. Image analysis was performed using the Amersham-Imager 680, ver. 2.0. software (https://www.cytivalifesciences.com/). Signal was quantified with GraphPad Prism 5, ver. 5.01. software (https://www.graphpad.com/). Cropped gels were displayed and full-length blots/gels are presented in supplementary Fig. S2.

To assess the effect of HMGB1 on 5-LO activity in VSMCs, LTB4 production was measured in cells stimulated with various concentrations (0–100 ng/ml) of HMGB1. An increased production of LTB4 was observed in VSMCs stimulated with HMGB1 at concentrations of 30 ng/ml and 100 ng/ml. In VSMCs stimulated with 30 ng/ml HMGB1, LTB4 production was increased at 12 h after stimulation, and then maintained up to 24 h. Likewise, at 12–24 h of HMGB1 stimulation, 5-LO expression was markedly increased in HMGB1-treated cells at concentrations of 30 ng/ml and 100 ng/ml (Fig. 2 and Supplementary Fig. S2).

### Role of 5-LO in HMGB1-induced MCP-1 expression in VSMCs

**Figure 3:**
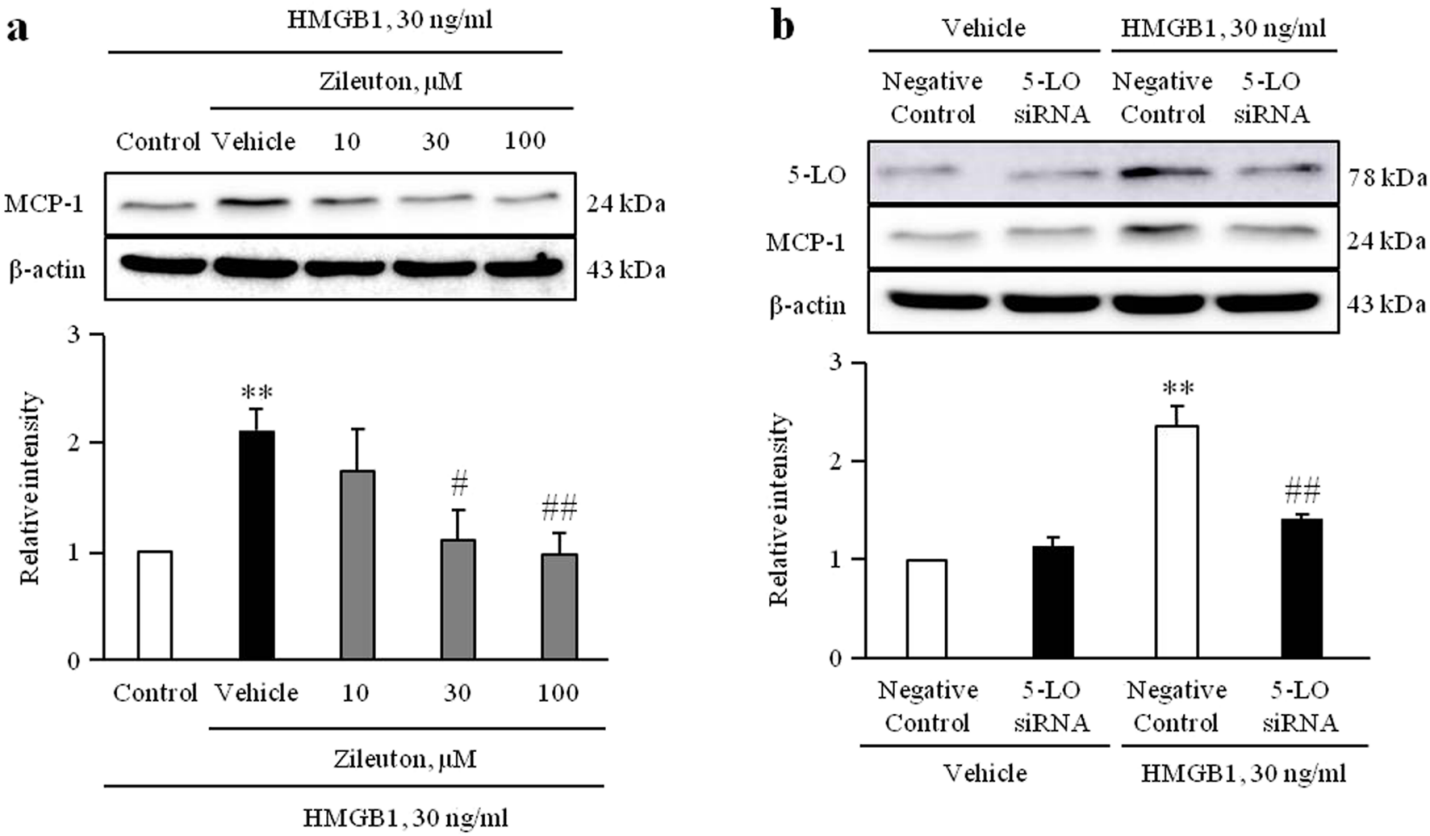
Role of 5-LO in HMGB1-induced MCP-1 expression in VSMCs. (**a**) VSMCs were pretreated with zileuton (0 to 100 μM) for 1 h, and then stimulated with HMGB1 (30 ng/ml) for 48 h. The expression levels of MCP-1 were determined by Western blotting using β-actin as an internal control. Results were expressed as the mean ± SEM of 4 independent experiments. **p < 0.01 vs. corresponding value in control and ^#^*p* < 0.05 and ^##^*p* < 0.01 vs corresponding value in vehicle. (**b**) VSMCs were transfected with 5-LO siRNA (200 nM) for 48 h, and then stimulated with HMGB1 (30 ng/ml) for 48 h. The expression levels of MCP-1 were determined by Western blotting using β-actin as an internal control. Results were expressed as the mean ± SEM of 3 independent experiments. **p < 0.01 vs. corresponding value in negative control in Vehicle and ^##^*p* < 0.01 vs corresponding value in negative control in HMGB1 group. Image analysis was performed using the Amersham-Imager 680, ver. 2.0. software (https://www.cytivalifesciences.com/). Signal was quantified with GraphPad Prism 5, ver. 5.01. software (https://www.graphpad.com/). Cropped gels were displayed and full-length blots/gels are presented in supplementary Fig. S3.

To evaluate the role of 5-LO signaling in HMGB1-induced MCP-1 expression, we measured MCP-1 expression in VSMCs pretreated with zileuton, a 5-LO inhibitor. As shown in Fig. 3a and Supplementary Fig. S3a, HMGB1-induced MCP-1 expression was significantly attenuated in cells pretreated with zileuton in a dose-dependent manner. Likewise, HMGB1-induced MCP-1 expression was also attenuated in cells depleted of 5-LO using siRNA (200 nM) (Fig. 3b and Supplementary Fig. S3b).

### Exogenous LTB4 increases MCP-1 expression in VSMCs

**Figure 4:**
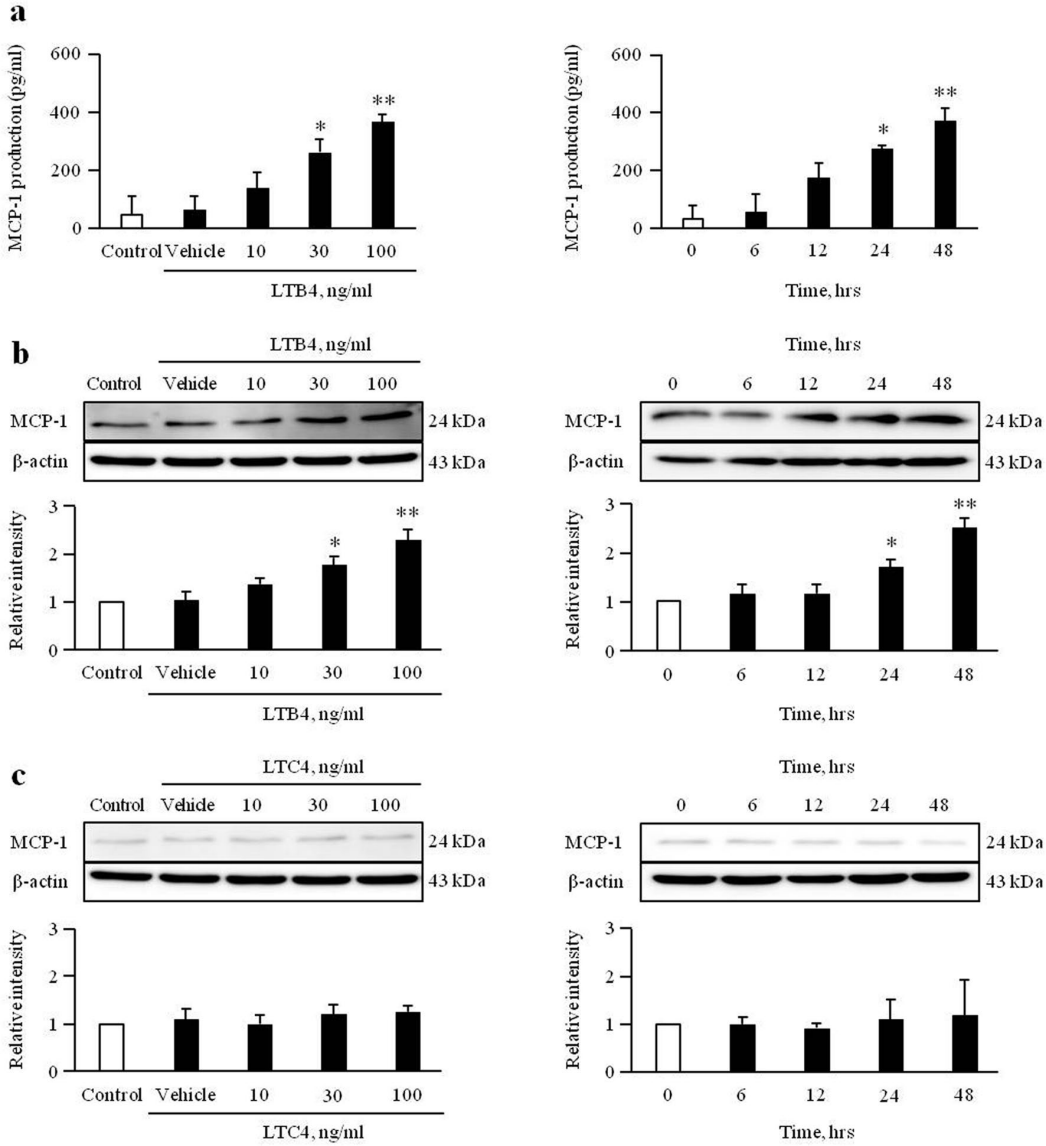
Role of LTB4 and LTC4 on MCP-1 production and expression in VSMCs. VSMCs were treated with LTB4 (0 to 100 ng/ml) for 48 h, or LTB4 (100 ng/ml) for 0 to 48 h. (**a**) MCP-1 production was measured in the cell culture media, and results were expressed as the mean ± SEM of 3 independent experiments. *p < 0.05 and **p < 0.01 vs. corresponding value at control or 0 h. (**b**) The expression levels of MCP-1 were determined by Western blotting using β-actin as an internal control. Results were expressed as the mean ± SEM of 4–5 independent experiments. *p < 0.05 and **p < 0.01 vs. corresponding value at control or 0 h. (**c**) VSMCs were treated with LTC4 (0 to 100 ng/ml) for 48 h, or LTC4 (100 ng/ml) for 0 to 48 h. The expression levels of MCP-1 were determined by Western blotting using β-actin as an internal control. Results were expressed as the mean ± SEM of 3 independent experiments. Image analysis was performed using the Amersham-Imager 680, ver. 2.0. software (https://www.cytivalifesciences.com/). Signal was quantified with GraphPad Prism 5, ver. 5.01. software (https://www.graphpad.com/). Cropped gels were displayed and full-length blots/gels are presented in supplementary Fig. S4.

As shown in Fig. 4a, MCP-1 production in LTB4 (100 ng/ml)-treated cells was gradually increased for up to 48 h, following which a dose-dependency was observed up to 100 ng/ml. To determine the effects of LTB4 on MCP-1 expression in VSMCs, cells were stimulated with LTB4 (100 ng/ml) for 48 h. In this study, MCP-1 expression was increased by LTB4 treatment in dose- and time-dependent manners (Fig. 4b, Supplementary Fig. S4a, and Supplementary Fig. S7a). However, MCP-1 expression was not induced in cells treated with LTC4 (Fig. 4c, Supplementary Fig. S4b, and Supplementary Fig. S7b).

### Role of BLTR1 signaling on LTB4-induced MCP-1 expression in VSMCs

**Figure 5:**
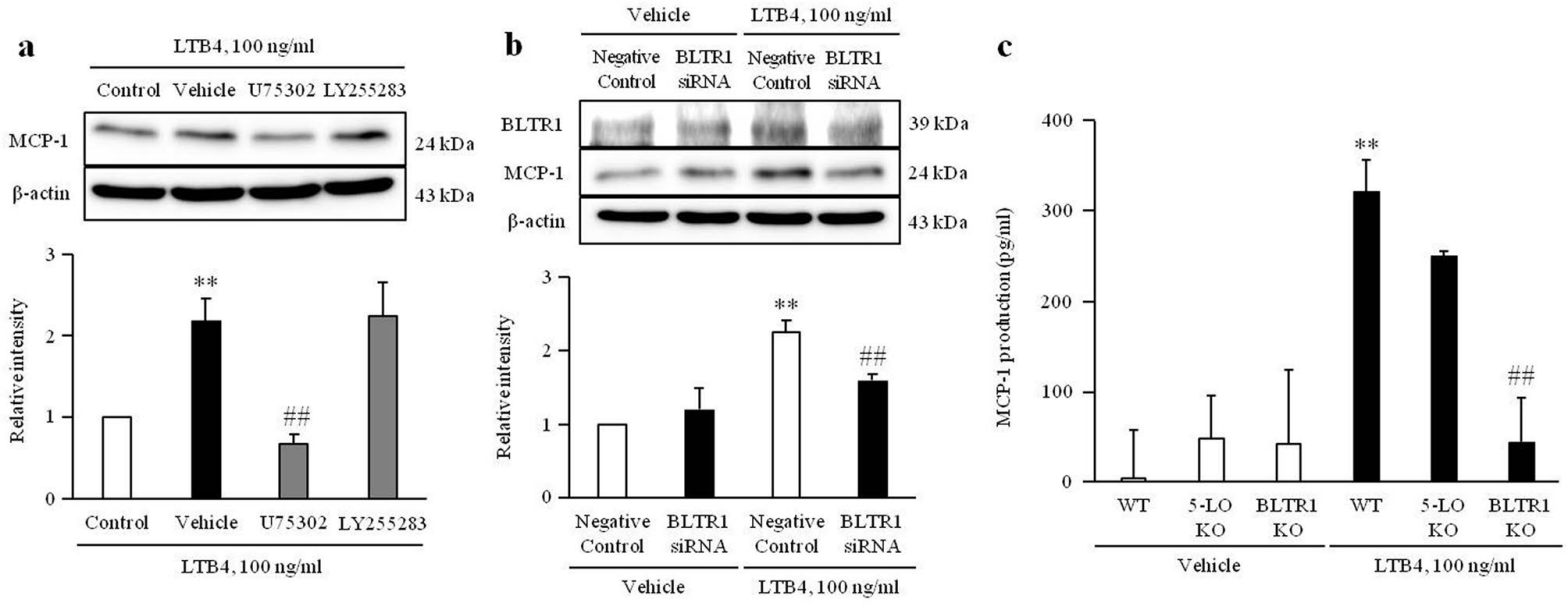
A pivotal role of BLTR1 on LTB4-induced MCP-1 production and expression in VSMCs. (**a**) VSMCs were pretreated with U75302 (10 μM) or LY255283 (10 μM) for 1 h, and then stimulated with LTB4 (100 ng/ml) for 48 h. The expression levels of MCP-1 were determined by Western blotting using β-actin as an internal control. Results were expressed as the mean ± SEM of 3 independent experiments. **p < 0.01 vs. value in control and ^##^*p* < 0.01 vs value in vehicle. (**b**) VSMCs were transfected with BLTR1 siRNA (200 nM) for 48 h, and then stimulated with LTB4 (100 ng/ml) for 48 h. The expression levels of MCP-1 were determined by Western blotting using β-actin as an internal control. Results were expressed as the mean ± SEM of 4 independent experiments. **p < 0.01 vs. corresponding negative control in Vehicle and ^##^*p* < 0.01 vs negative control in LTB4 group. Image analysis was performed using the Amersham-Imager 680, ver. 2.0. software (https://www.cytivalifesciences.com/). Cropped gels were displayed and full-length blots/gels are presented in supplementary Fig. S5. (**c**) VSMCs cultured from thoracic aorta of WT, 5-LO- and BLTR1-KO mice were treated with LTB4 (100 ng/ml) for 48 h. MCP-1 production was measured in the cell culture media, and results were expressed as the mean ± SEM of 3 independent experiments. **p < 0.01 vs. corresponding value in vehicle and ^##^*p* < 0.01 vs WT in LTB4 group. Signal was quantified with GraphPad Prism 5, ver. 5.01. software (https://www.graphpad.com/).

To identify leukotriene receptor signaling involved in LTB4-induced MCP-1 expression, VSMCs were stimulated with LTB4 (100 ng/ml) in the presence or absence of LTB4 receptor antagonists. As shown in Fig. 5a and Supplementary Fig. S5a, LTB4-induced MCP-1 expression was inhibited by pretreatment with U75302 (10 μM), a BLT1 receptor antagonist, but not by LY255283 (10 μM), a BLT2 receptor antagonist. As expected, LTB4-induced expression of MCP-1 was markedly attenuated in BLTR1-depleted cells using siRNA (200 nM) (Fig. 5b and Supplementary Fig. S5b). Moreover, HMGB1-induced MCP-1 production was markedly attenuated in BLTR1-deficient cells cultured from thoracic aorta of BLTR1-KO mice, but not in 5-LO-deficient cells cultured from thoracic aorta of 5-LO-KO mice (Fig. 5c), suggesting a pivotal involvement of BLTR1 in LTB4-induced MCP-1 production.

### A pivotal role of BLTR1 signaling on HMGB1-induced MCP-1 production in VSMCs

**Figure 6:**
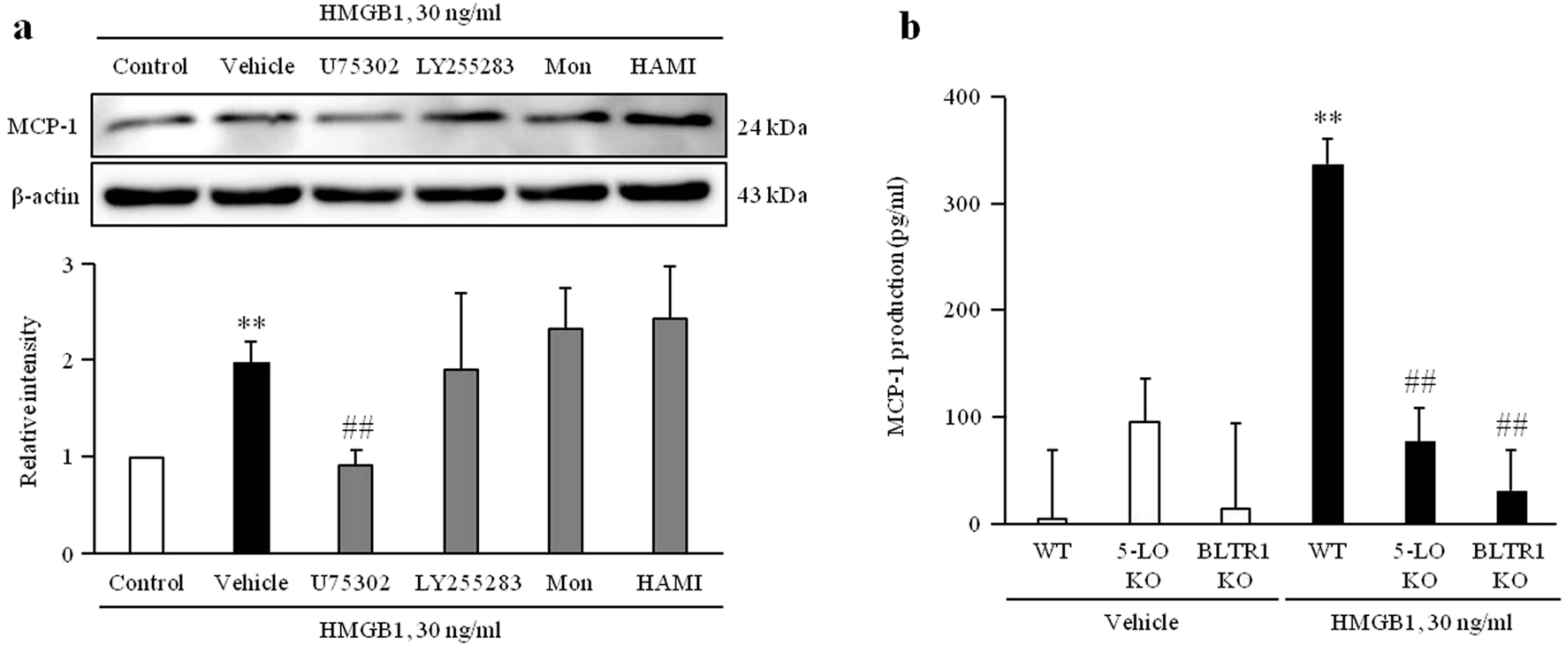
An essential role of BLTR1 on HMGB1-induced MCP-1 production and expression in VSMCs. (**a**) VSMCs were pretreated with U75302 (10 μM), LY255283 (10 μM), montelukast (1 μM) and HAMI3379 (10 μM) for 1 h, and then stimulated with HMGB1 (30 ng/ml) for 48 h. The expression of MCP-1 was determined by Western blotting using β-actin as an internal control. Results were expressed as the mean ± SEM of 4 independent experiments. **p < 0.01 vs. control and ^##^*p* < 0.01 vs vehicle. Image analysis was performed using the Amersham-Imager 680, ver. 2.0. software (https://www.cytivalifesciences.com/). Cropped gels were displayed and full-length blots/gels are presented in supplementary Fig. S6. (**b**) VSMCs cultured from thoracic aorta of WT, 5-LO- and BLTR1-KO mice were treated with HMGB1 (30 ng/ml) for 48 h. MCP-1 production was measured in the cell culture media, and results were expressed as the mean ± SEM of 3 independent experiments. **p < 0.01 vs. corresponding value in Vehicle and ^##^*p* < 0.01 vs WT in HMGB1 group. Signal was quantified with GraphPad Prism 5, ver. 5.01. software (https://www.graphpad.com/).

To identify leukotriene receptor signaling involved in HMGB1-induced MCP-1 expression, VSMCs were stimulated with HMGB1 (30 ng/ml) in the presence or absence of LTB4 receptor antagonists. As shown in Fig. 6a and Supplementary Fig. S6, MCP-1 expression induced by HMGB1 was significantly inhibited by pretreatment with a BLTR1 antagonist, U75302 (10 μM), but not BLTR2 and CysLTRs antagonists, including LY255283 (10 μM), montelukast (1 μM) and HAMI3379 (10 μM). Likewise, MCP-1 production induced by HMGB1 was significantly inhibited by pretreatment with a BLTR1 antagonist, U75302 (10 μM) in human VSMCs (Supplementary Fig. S7d). The increased MCP-1 production in HMGB1-stimulated VSMC was markedly attenuated in 5-LO-deficient cells as well as in BLTR1-deficient cells (Fig. 6b), indicating a pivotal role of LTB4-BLTR1 signaling in MCP-1 expression in VSMCs.

## Discussion

This study shows that the expression of MCP-1 was increased in VSMCs stimulated with HMGB1, which was attenuated in 5-LO-deficient cells as well as in cells treated with a 5-LO inhibitor. In contrast, exogenous LTB4, but not LTC4, increased MCP-1 expression in 5-LO-deficient cells. Moreover, the increased MCP-1 expression in cells stimulated with LTB4 and HMGB1 was attenuated in cells treated with U75302, a BLTR1 inhibitor as well as in BLTR1-deficient cells, indicating a pivotal role of 5-LO-derived LTB4 and BLTR1 signaling in MCP-1 expression in VSMCs.

Inflammation of the vasculatures is a key event in the initiation of various vascular diseases including atherosclerosis and angioplasty-induced vascular remodeling. In the injured vasculatures, inflammatory cell migration and infiltration into the damaged tissues are followed by increased production of endogenous DAMPs^23,24^. Among various injury-induced mediators, HMGB1 is one of the best characterized DAMPs in the development and progression of cardiovascular diseases^25–27^. Via activation of NLR family pyrin domains containing protein 3 (NLRP3) inflammasome in VSMCs, HMGB1 increases the production of inflammatory cytokines, including IL-1β^3^, which enhances VSMC proliferation and migration through regulation of HMGB1^28^. Thus, HMGB1 released in the injured vasculatures is suggested to be a key player in the pathogenesis of vascular inflammation and remodeling. However, the precise role of HMGB1 and VSMCs in the initiation of injury-mediated vascular inflammation is unclear.

In inflammatory processes in the injured vasculatures, MCP-1 signaling plays an important role via recruitment of monocytes, memory T cells, and dendritic cells to the sites of tissue injury^19,29^. MCP-1, a proinflammatory cytokine, is one of the most potent chemoattractant agents for monocytes, and promotes vascular remodeling by enhancing VSMC migration and proliferation^30^. Moreover, MCP-1 induces the transmigration of circulating monocytes and exerts various effects on monocytes, including cytokine production and adhesion molecule expression^31^. Previously, elevated levels of MCP-1 were reported in patients with myocardial infarction^32^ and systemic inflammation^33^. In addition, the increased MCP-1 expression was detected in atherosclerotic lesions but not in normal arteries^34^, indicating an active role of MCP-1 in the process of vascular inflammation in the injured vasculatures. Reportedly, HMGB1 produces different concentrations of MCP-1 in various cell types including human monocytes (0–1000 pg/ml), PBMCs (0–100 ng/ml)^35^, and endothelial cells (1–100 ng/ml)^36^. In murine peritoneal macrophages, HMGB1 (0–5000 ng/ml) increased MCP-1 production up to 300 pg/ml^24^, indicating various concentrations of MCP-1 is produced in a variety of cell types stimulated with HMGB1. Considering the previous reports that low concentrations (1–100 pg/ml) of rat MCP-1 directly promote VSMC proliferation and migration in the course of pathological events^37^, it is suggested that MCP-1 produced by murine VSMCs plays an important role in vascular inflammation and remodeling. Although MCP-1 is considered as a key factor that initiates vascular inflammation^38^, its cellular source and molecular mechanisms involved in its production in the injured vasculatures remain unclear.

In the present study, the expression and production of MCP-1 were markedly increased in VSMCs exposed to HMGB1. The importance of 5-LO-derived mediators in cellular responses mediated by inflammatory agents has been described previously^39,40^. In our previous study, 5-LO signaling in VSMCs was reported to play a key role in mediating vascular effects of HMGB1^41,42^, a major DAMP related to various vascular diseases^43^. Another study also showed that disruption of the LTB4 signaling pathway in monocytes restrains several elements of injury response, indicating that LTB4-induced MCP-1 expression in human monocytes plays a critical role in the development of vascular inflammation^11^. To further investigate the active role of 5-LO signaling in MCP-1 production in the injured vasculatures, we determined the role of 5-LO signaling in MCP-1 expression and production in VSMCs exposed to HMGB1. Our results demonstrated that the expression of MCP-1 and 5-LO was markedly increased in HMGB1-stimulated VSMCs. The HMGB1-induced MCP-1 expression was markedly attenuated by inhibition of 5-LO, indicating an active involvement of 5-LO signaling in HMGB1-induced MCP-1 expression in VSMCs.

LTB4, a potent proinflammatory mediator, plays an important role in the progression of vascular inflammatory diseases including atherosclerosis^6,44^. The biological effects of LTB4 are mainly mediated by the activation of a LTB4 receptor 1 (BLTR1) and a LTB4 receptor 2 (BLTR2)^45^. Reportedly, the binding of the high-affinity receptor BLT1 and the low-affinity receptor BLT2 to their ligands has been implicated in vascular remodeling^10,11^. In this study, both exogenous LTB4 and HMGB1 induced MCP-1 expression and production in VSMCs. To further investigate the role of LTB4 signaling in HMGB1-induced MCP-1 expression, VSMCs were stimulated with HMGB1 in the presence of various inhibitors for leukotriene receptors, including LTB4 receptors or LTC4 receptors. Results showed that treatment with U75302, a BLTR1 inhibitor, exclusively attenuated MCP-1 production. Moreover, the role of BLTR1 in HMGB1-induced MCP-1 production in VSMCs was demonstrated using BLTR1-deficient cells and cells treated with U75302, a BLTR1 inhibitor. Interestingly, in contrast to the increased MCP-1 expression in 5-LO-deficient cells treated with LTB4, MCP-1 production in 5-LO-deficient VSMCs stimulated with HMGB1 was not increased. Moreover, the MCP-1 expression induced in cells stimulated LTB4 and HMGB1 was attenuated in BLTR1-deficient cells, indicating a pivotal role for 5-LO-derived LTB4 and BLTR1 signaling in MCP-1 expression in VSMCs.

Taken together, our data suggest that 5-LO-derived LTB4 produced by HMGB1-stimulated VSMCs increased MCP-1 expression in VSMCs of the injured vasculatures via BLTR1 signaling. Thus, the LTB4-BLTR1 signaling axis in VSMCs might serve as a potential therapeutic target for vascular inflammation in the injured vasculatures.

## Supplementary Information

Supplementary Information.
