# Supplementary material for: 5-LO-derived LTB4 plays a key role in MCP-1 expression in HMGB1-exposed VSMCs via a BLTR1 signaling axis
Source: Sci Rep. 2021 May 27;11:11100. doi: 10.1038/s41598-021-90636-2 (PMC8160259; doi:10.1038/s41598-021-90636-2)
Supplement: Supplementary file 1 — Supplementary Information. [file 41598_2021_90636_MOESM1_ESM.pdf]

5-LO-derived LTB4 plays a key role in MCP-1 expression in HMGB1-exposed VSMCs via a BLTR1 signaling axis

Jong Min Choi[1,2,#], Seung Eun Baek[1,2,#], Ji On Kim[1,2], Eun Yeong Jeon[1,2], Eun Jeong Jang[1,2], Chi Dae Kim[1,2,3*]

[1]Department of Pharmacology, School of Medicine, Pusan National University, Yangsan, Gyeongnam 50612, Republic of Korea.

[2]Gene & Cell Therapy Research Center for Vessel-associated Diseases, Pusan National University, Yangsan, Gyeongnam 50612, Republic of Korea.

[3]Research Institute for Convergence of Biomedical Science and Technology, Pusan National University Yangsan Hospital, Gyeongnam 50612, Republic of Korea.

[#] Jong Min Choi and Seung Eun Baek equally contributed to this study.

[*] **Corresponding author:** Chi Dae Kim, MD, PhD.

Department of Pharmacology, School of Medicine, Pusan National University, Yangsan, Gyeongnam 50612, Republic of Korea.

Tel: +82 51 510 8063, Fax: +82 51 510 8068, E-mail : chidkim@pusan.ac.kr

Materials and methods

- Chemicals and antibodies

Recombinant human HMGB1 antibody was purchased from R&D System Inc. (Minneapolis, MN, USA). Leukotriene C4 (LTC4), Leukotriene B4 (LTB4), LTB4 receptor 1 (BLTR1) inhibitor (U75302) and BLTR2 inhibitor (LY255283) were purchased from Cayman Chemical Inc (Ann Arbor, MI, USA).

- Cell culture

Human VSMCs were purchased from ATCC (Manassas, VA, USA) and cultured in a cell culture dish containing smooth muscle cell growth medium (Gibco BRL) with smooth muscle growth supplement (Gibco BRL), 10% FBS and antibiotic-antimycotic solution (Gibco BRL) at 37℃ (1).

- Enzyme-linked immunosorbent assay (ELISA)

The cultured human VSMCs pretreated with U75302 and LY255283, and then stimulated with HMGB1. MCP-1 production in the cell culture media was measured using a human MCP-1 ELISA kit (R&D System) according to manufacturer's protocols. The plate was read at 450 nm.

- Measurement of mRNA expression

MCP-1 mRNA expressions in human VSMCs were quantified by reverse transcription polymerase chain reaction (RT-PCR) using GAPDH mRNA as an internal standard. Total RNA was isolated from cultured cells using Qiazol (Qiagen, Hilden, Germany). All cDNA reverse transcribed by RNA (1 μg) using the ImProm-II Reverse Transcription System (Promega, Madison, WI). PCR amplification was performed using specific primers at MCP-1 (forward, 5`-ATG CAG TTA ATG CCC CAC TC-3` ;

reverse, 5`-TTC CTT ATT GGG GTC AGC AC-3`) and GAPDH (forward, 5`-GAG TCA ACG GAT TTG GTC GT-3` ; reverse, 5`-TGT GGT CAT GAG TCC TTC CA -3`) (2). Gels were detected using Syngene GVM20 UV transilluminator and utilizing image capturing software (Canon Utilities ZoomBrowser EX 5.0, version. 5.0.0.142.).

- Statistical analysis

Results were expressed as means ± SEM. One-way analysis of variance (ANOVA) followed by Dunnett's or Tukey's multiple comparison tests were used to determine the significances of experimental results. Analysis of data was quantified using GraphPad Prism 5, version. 5.01. software (GraphPad Software, USA). Statistical significance was accepted for p values < 0.05.

Supplementary Figures

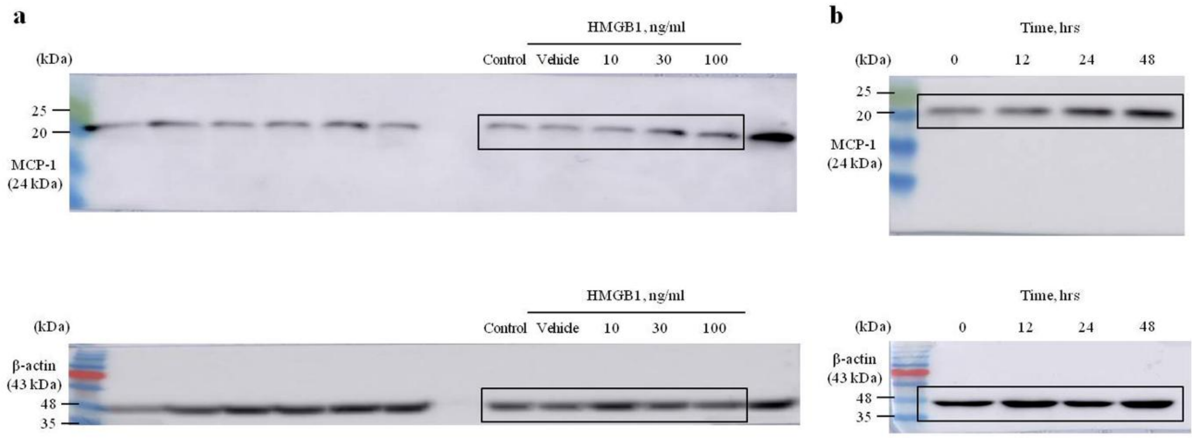

Supplementary Figure S1. Full-length blots/gel of MCP-1 and β-actin in VSMCs of treated HMGB1.

Rat aortic VSMCs were treated with HMGB1 (0 to 100 ng/ml) for 48 hrs (a) or HMGB1 (30 ng/ml) for 0 to 48 hrs (b). Boxed region shows the cropped region used in the main manuscript. Image was captured using the Amersham-Imager 680, ver. 2.0. software (https://www.cytivalifesciences.com/).

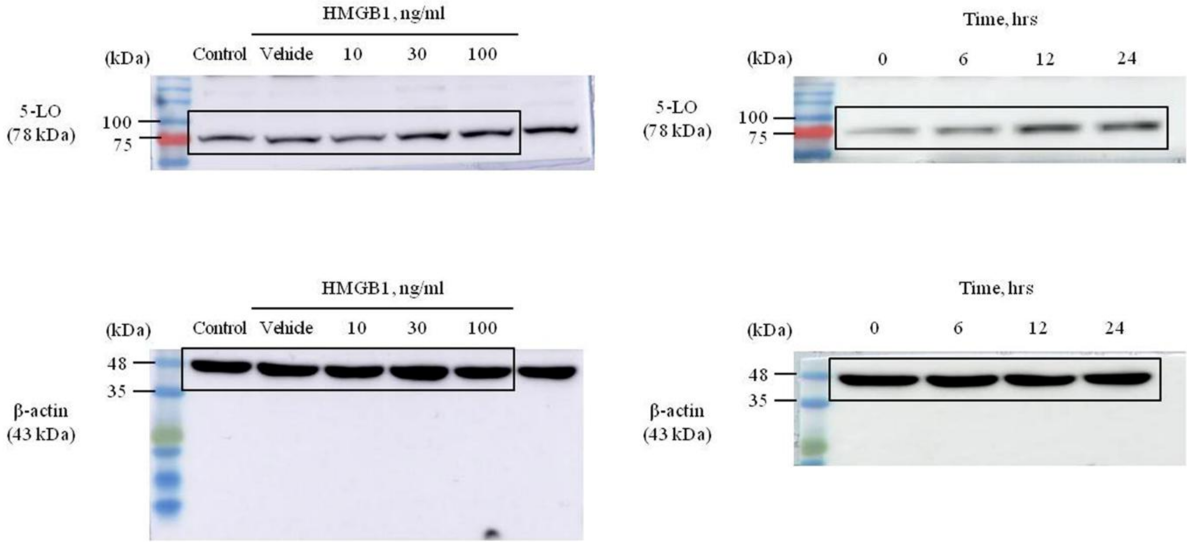

Supplementary Figure S2. Full-length blots/gel of 5-LO and β-actin in VSMCs of treated HMGB1.

Rat aortic VSMCs were treated with HMGB1 (0 to 100 ng/ml) for 12 hrs or HMGB1 (30 ng/ml) for 0 to 24 hrs. Boxed region shows the cropped region used in the main manuscript. Image was captured using the Amersham-Imager 680, ver. 2.0. software (https://www.cytivalifesciences.com/).

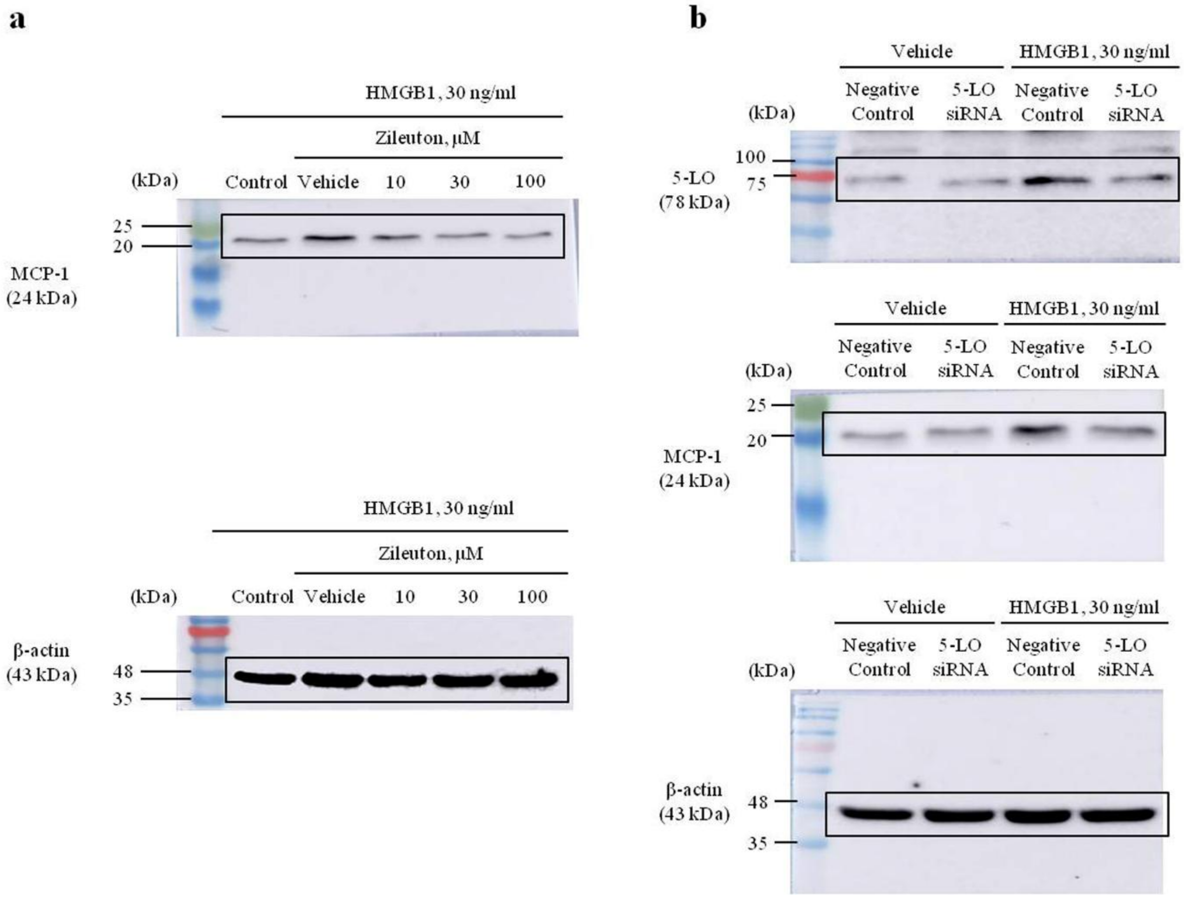

Supplementary Figure S3. Full-length blots/gel of 5-LO, MCP-1 and β-actin in VSMCs of treated HMGB1.

(a) VSMCs were pretreated with zileuton (0 to 100 μM) for 1 hr, and then stimulated with HMGB1 (30 ng/ml) for 48 hrs. (b) VSMCs were transfected with 5-LO siRNA (200 nM) for 48 hrs, and then stimulated with HMGB1 (30 ng/ml) for 48 hrs. Boxed region shows the cropped region used in the main manuscript. Image was captured using the Amersham-Imager 680, ver. 2.0. software (https://www.cytivalifesciences.com/).

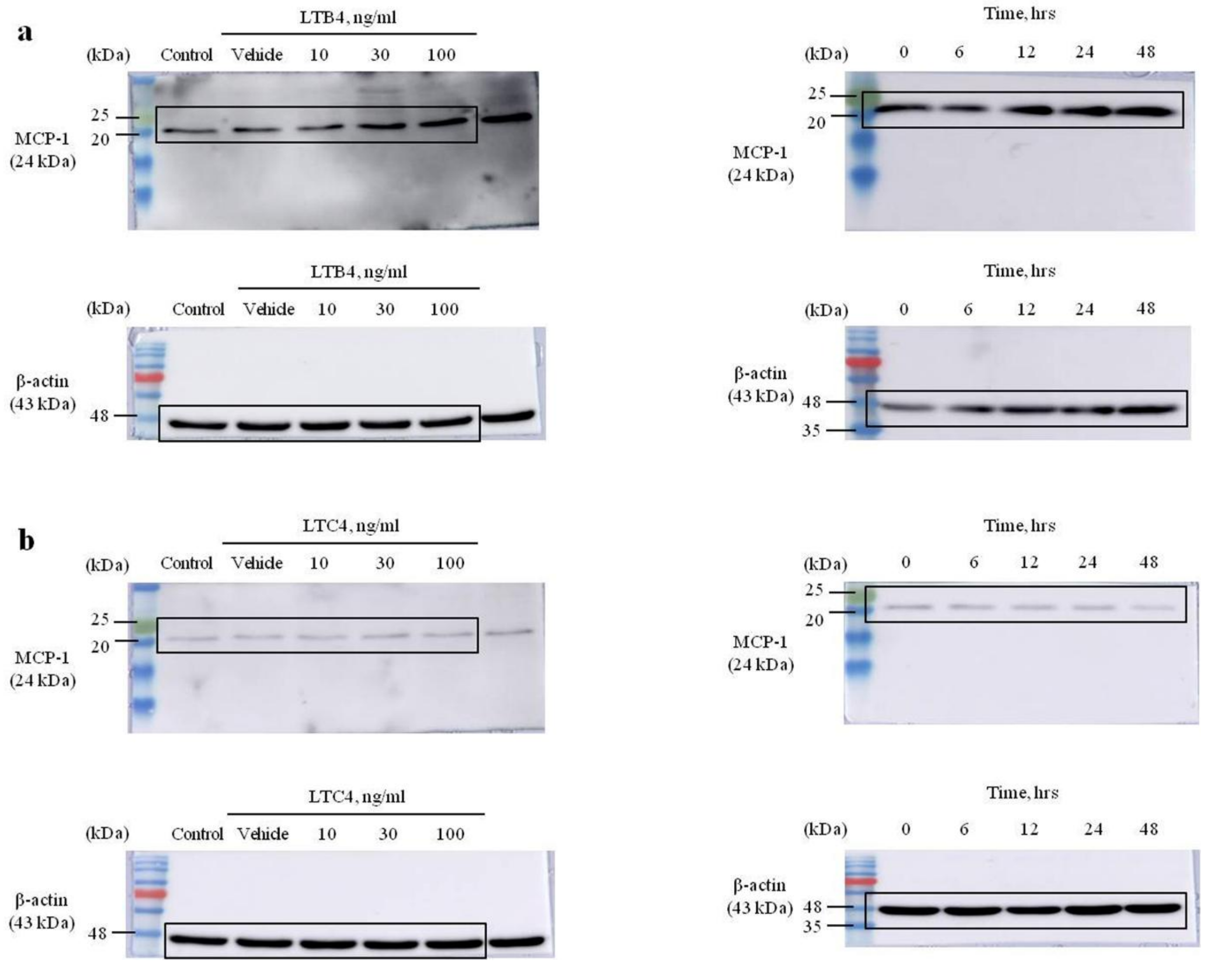

Supplementary Figure S4. Full-length blots/gel of MCP-1 and β-actin in VSMCs of treated LTs.

(a) VSMCs were treated with LTB4 (0 to 100 ng/ml) for 48 hrs, or LTB4 (100 ng/ml) for 0 to 48 hrs.

(b) VSMCs were treated with LTC4 (0 to 100 ng/ml) for 48 hrs, or LTC4 (100 ng/ml) for 0 to 48 hrs. Boxed region shows the cropped region used in the main manuscript. Image was captured using the Amersham-Imager 680, ver. 2.0. software (https://www.cytivalifesciences.com/).

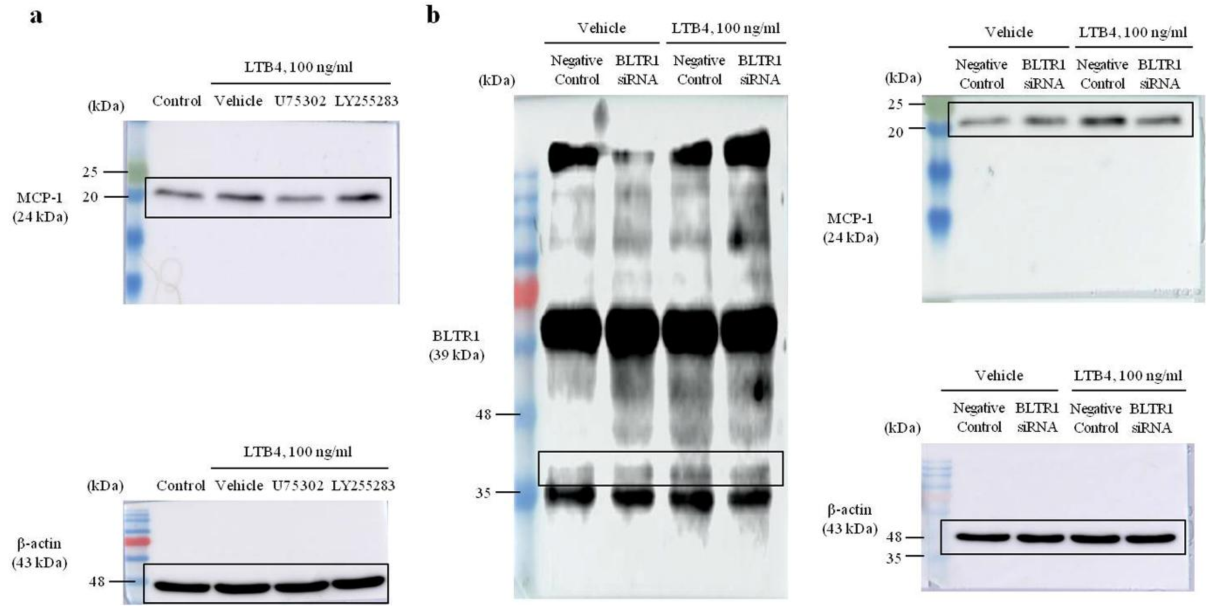

Supplementary Figure S5. Full-length blots/gel of MCP-1, BLTR1 and β-actin in VSMCs of treated LTB4.

(a) VSMCs were pretreated with U75302 (10 μM) or LY255283 (10 μM) for 1 hr, and then stimulated with LTB4 (100 ng/ml) for 48 hrs. (b) VSMCs were transfected with BLTR1 siRNA (200 nM) for 48 hrs, and then stimulated with LTB4 (100 ng/ml) for 48 hrs. Boxed region shows the cropped region used in the main manuscript. Image was captured using the Amersham-Imager 680, ver. 2.0. software (https://www.cytivalifesciences.com/).

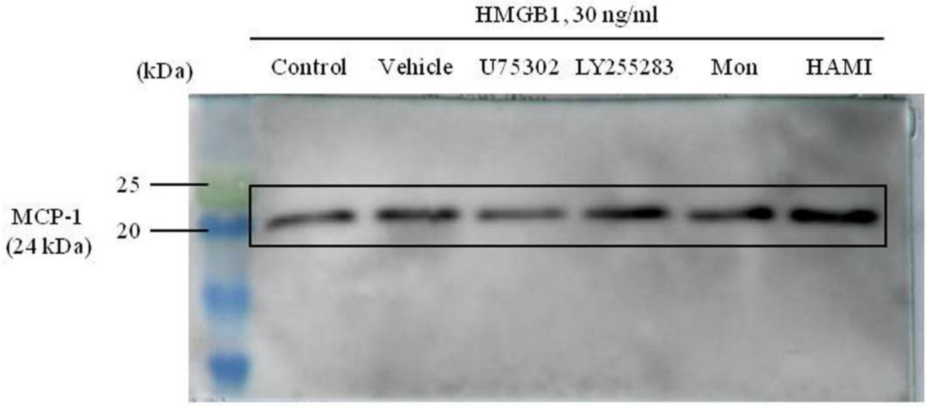

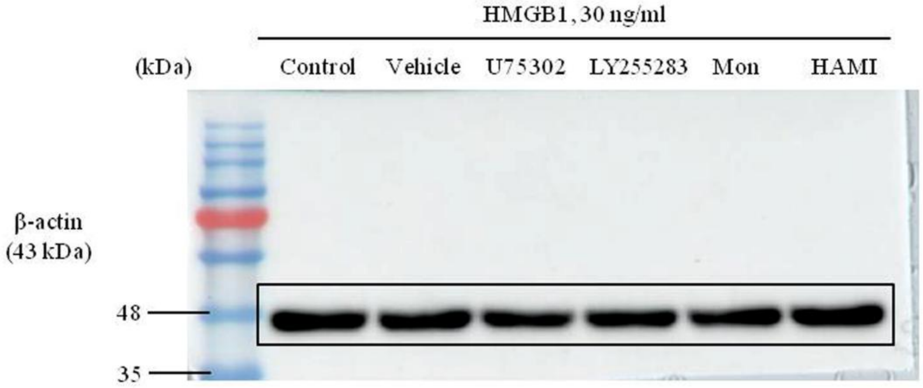

Supplementary Figure S6. Full-length blots/gel of MCP-1 and β-actin in VSMCs of treated HMGB1.

VSMCs were pretreated with U75302 (10 μM), LY255283 (10 μM), montelukast (1 μM) and HAMI3379 (10 μM) for 1 hr, and then stimulated with HMGB1 (30 ng/ml) for 48 hrs. Boxed region shows the cropped region used in the main manuscript. Image was captured using the Amersham-Imager 680, ver. 2.0. software (https://www.cytivalifesciences.com/).

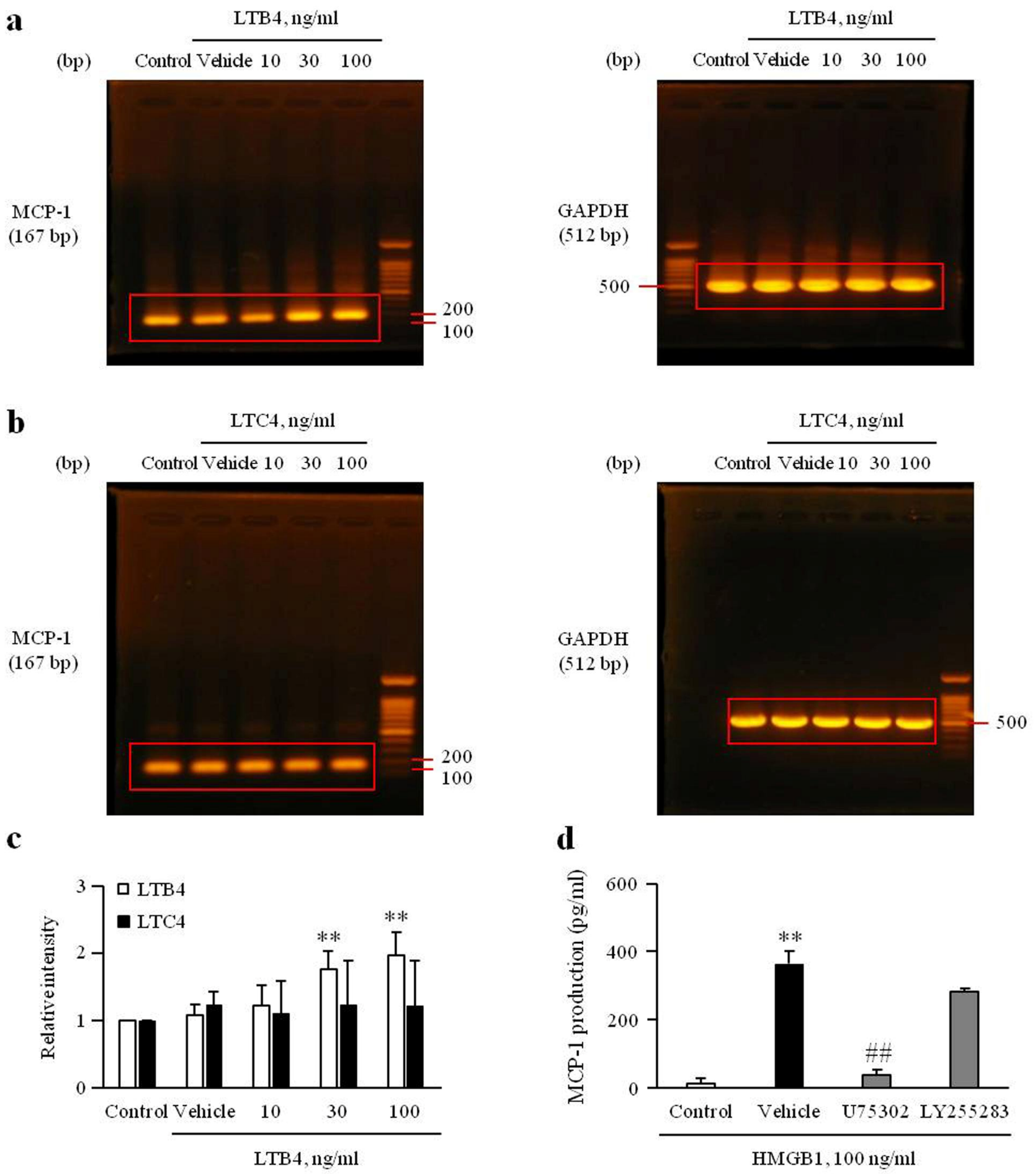

Supplementary Figure S7. Effects of LTs and HMGB1 on MCP-1 expression and production in human VSMCs

(a) Human aortic VSMCs were treated with LTB4 (0 to 100 ng/ml) for 12 hrs. The mRNA levels of MCP-1 were determined by RT-PCR and GAPDH was used as a control. (b) Human aortic VSMCs were treated with LTC4 (0 to 100 ng/ml) for 12 hrs. The mRNA levels of MCP-1 were determined by RT-PCR and GAPDH was used as a control. Image analysis was performed using the Canon Utilities

ZoomBrowser EX 5.0, ver. 5.0.0.142. software (https://global.canon/). (c) Relative intensities were expressed as the means ± SEMs of 4 independent experiments. $^{**}P < 0.01$ corresponding value at control. (d) Human VSMCs were pretreated with U75302 (10 μM) or LY255283 (10 μM) for 1 hr, and then stimulated with HMGB1 (100 ng/ml) for 48 hrs. MCP-1 production was measured in the cell culture media, and result was expressed as the mean ± SEM of 3 independent experiments. $^{**}P < 0.01$ vs. corresponding value in Control and $^{\#\#}P < 0.01$ vs Vehicle. Signal was quantified with GraphPad Prism 5, ver. 5.01. software (https://www.graphpad.com/).

References

1. Jang, E. J., Baek, S. E., Kim, E. J., Park, S. Y. & Kim, C. D. HMGB1 Enhances AGE-mediated VSMC Proliferation via an Increase in 5-LO-linked RAGE Expression. *Vascul Pharmacol* **118-119,** 106559, https://doi.org/10.1016/j.vph.2019.04.001 (2019).

2. Jang. M. A. *et al.* α-Iso-Cubebene Inhibits PDGF-Induced Vascular Smooth Muscle Cell Proliferation by Suppressing Osteopontin Expression. *PLoS One* **12**, e0170699, https://doi.org/10.1371/journal.pone.0170699 (2017).
